# Sodium carboxymethyl cellulose coating pretreatment combined with multi-frequency ultrasound assisted vacuum far-infrared drying: An emerging approach to enhance drying characteristics, physicochemical properties, and sensory attributes of *Cornus officinalis*

**DOI:** 10.1016/j.ultsonch.2025.107657

**Published:** 2025-10-28

**Authors:** Zepeng Zang, Xiaopeng Huang, Guojun Ma, Fangxin Wan, Xiaoping Yang, Qiaozhu Zhao, Yanrui Xu, Fei Dai

**Affiliations:** College of Mechanical and Electrical Engineering, Gansu Agricultural University, Lanzhou 730070, China

**Keywords:** *Cornus officinalis*, Multi-frequency ultrasound, Vacuum far-infrared drying, Sodium carboxymethyl cellulose pretreatment, Physicochemical properties

## Abstract

To enhance the drying efficiency and improve the sensory quality of *Cornus officinalis*, this study investigated the effects of sodium carboxymethyl cellulose (CMC-Na) coating combined with multi-frequency ultrasound assisted vacuum far-infrared (MFUS-VFIR) drying on its drying characteristics, physicochemical properties, and sensory attributes. Application of multi-frequency ultrasound (MFUS) during VFIR dehydration shortened the drying time by 12.12–39.39 % and increased the average drying rate by 15.38–69.23 % compared with VFIR alone. Physicochemical analyses revealed that the (MFUS-VFIR)-20/28/40 kHz treatment yielded dried products with higher retention of total phenolics, natural bioactive compounds, organic acids, total carotenoids, ascorbic acid, soluble solids, and total flavonoids, along with superior color quality. Under these conditions, antioxidant capacity increased by 10.89–23.68 %, 14.41–25.91 %, and 7.10–58.32 %, respectively, relative to dual-frequency ultrasound treatments. Scanning electron microscopy showed that MFUS treatment produced distinct honeycomb-like pores with larger apertures compared with SFUS, indicating reduced surface cracking and expanded micro-channels for mass transfer, thereby lowering mass transfer resistance. The overall sensory acceptability of (MFUS-VFIR)-20/28/40 kHz dried products reached 8.50, representing a 41.67 % and 13.33–30.77 % improvement over VFIR and SFUS-VFIR samples (*P* < 0.05), with lower bitterness and off-flavor scores. Principal component analysis (PCA), hierarchical cluster analysis (HCA), and correlation network heat mapping revealed that MFUS-treated samples clustered closely in multidimensional quality space and exhibited significant positive correlations with antioxidant activity, physicochemical quality, and flavor retention. Notably, the energy consumption of (MFUS-VFIR)-20/28/40 kHz treatment was 88.68 kW·h·kg^−1^, slightly higher than that of the control and SFUS-VFIR treatments. These findings provide a scientific basis and technical reference for quality optimization, energy-efficient drying, and high-value utilization of *Cornus officinalis*.

## 1 Introduction

*Cornus officinalis* Sieb. et Zucc., belonging to the family *Cornaceae*, is widely utilized in both traditional medicine and the modern functional food industry due to its mature dried fruits, which are recognized as a medicinal and edible resource [1,2]. Currently, *Cornus officinalis* is mainly distributed in the temperate regions of East Asia, with large-scale cultivation in China, Korea, and Japan. Pharmacological studies have demonstrated that it is rich in bioactive compounds, including iridoids (e.g., loganin, morroniside), triterpenoids (e.g., ursolic acid, oleanolic acid), flavonoids, polyphenols, and organic acids [3,4]. These constituents exhibit notable activities such as antioxidant, hypoglycemic, neuroprotective, and immunomodulatory effects, indicating significant medicinal potential and broad application prospects. However, as a medicinal and edible plant, fresh *Cornus officinalis* has high moisture content and vigorous respiration post-harvest, making it highly susceptible to dehydration, browning, softening, microbial spoilage, and enzymatic degradation if not promptly processed, thereby compromising its physicochemical quality and economic value [5]. Therefore, an efficient and well-designed drying process is essential during primary processing, playing a critical role in prolonging shelf life, stabilizing active components, preserving sensory quality, and ensuring medicinal efficacy. Traditional drying methods, such as sun drying, is widely used in the primary processing of *Cornus officinalis* due to their operational simplicity and low cost. However, these methods suffer from prolonged drying time, low heat transfer efficiency, degradation of heat-sensitive compounds, and deterioration of product color, making them insufficient for producing high-quality products [6,7]. Moreover, due to the dense fruit tissue and complex moisture migration, traditional drying often leads to uneven dehydration, resulting in the “dry outside and wet inside” defect, which compromises product stability and rehydration capacity. Although advanced techniques such as vacuum drying [5,8], heat pump drying [9], microwave drying [10], and freeze drying [11] have been explored, their application is limited by high energy consumption, low efficiency, and poor industrial scalability. Therefore, developing a novel drying technology that ensures both efficient energy utilization and quality retention is essential for the high-quality development of the *Cornus officinalis*.

Sodium carboxymethyl cellulose (CMC-Na) coating, as an edible protective layer, exhibits excellent film-forming ability and moisture barrier properties [12]. During drying, it forms a uniform and dense encapsulation structure that mitigates tissue shrinkage or cracking caused by evaporation stress [13,14]. Furthermore, as a pretreatment, the CMC-Na coating effectively isolates oxygen and light, significantly inhibiting the oxidative degradation of organic acids, polyphenols, and other active compounds, thereby enhancing the color stability, flavor retention, and functional activity of the dried products [15]. Nevertheless, CMC-Na treatment may also impose adverse effects on the drying rate and dehydration time. At elevated concentrations, its film-forming property creates a dense barrier layer that impedes moisture diffusion, thereby extending the overall drying cycle and reducing both the drying rate and the mass transfer driving force. In addition, the coating’s barrier effect on heat transfer limits localized temperature rise, which consequently constrains moisture evaporation and retards the drying process. In this context, selecting an advanced drying technique in combination with CMC-Na coating is particularly critical. Vacuum far-infrared drying (VFIR), an advanced dehydration technology integrating radiative heating and low-pressure drying, has demonstrated superior performance in processing heat-sensitive agricultural products in recent years [16]. It employs far-infrared radiation to directly excite water molecules within the material, achieving volumetric heating, while the vacuum environment lowers the vaporization temperature, facilitating low-temperature dehydration and effectively suppressing quality-degrading reactions such as polyphenol oxidation and non-enzymatic browning [17]. However, for fruit-based medicinal materials with high pectin content and dense tissue structure, VFIR still faces challenges such as slow internal moisture diffusion and low heat and mass transfer efficiency, resulting in a marked decline in drying rate during the later stages [18]. To address these limitations, researchers have begun to investigate multi-physical field coupling strategies to improve heat and mass transfer efficiency and drying uniformity while preserving product quality, offering a novel approach for deep dehydration of heat-sensitive medicinal and edible materials.

In recent years, ultrasound-assisted treatment (US), as a green and non-thermal physical technology, has shown high application potential in processing heat-sensitive agricultural products and medicinal plants due to its significant advantages in enhancing heat and mass transfer, accelerating moisture migration, and alleviating internal diffusion resistance [19,20]. Its fundamental mechanisms include cavitation, microjet, mechanical vibration, and localized thermal effects induced during acoustic wave propagation. These physical effects can create microchannel structures within cellular tissues and disrupt the integrity of cell walls and membranes, thereby reducing boundary layer resistance and promoting internal moisture migration and release [21]. Additionally, ultrasound can effectively mitigate moisture migration resistance caused by intact cellular structures and tissue compactness, addressing the decline in drying rate during the mid to late stages of dehydration [22]. Single-frequency ultrasound (SFUS) has achieved favorable outcomes in the drying of berry fruits such as wolfberry [23] and ginger slices [24]. Relevant studies have shown that SFUS can reduce drying time by 15 %–30 % while significantly improving polyphenol content, antioxidant capacity, and color retention of the products. Nevertheless, SFUS is limited by its fixed frequency, often resulting in uneven acoustic field distribution and localized energy concentration. This restricts its penetration in complex and dense plant matrices, making it difficult to achieve effective and uniform internal enhancement [25]. Moreover, cavitation effects under a fixed frequency are highly variable and spatially confined, which may cause micro-damage to surface tissues, compromising structural stability and quality uniformity of the dried product [26]. Thus, insufficient spatial uniformity of acoustic energy transmission and low energy utilization efficiency have become major technical bottlenecks restricting the application of SFUS in agricultural product drying.

To overcome the above limitations, multi-frequency ultrasound (MFUS) drying has attracted increasing research attention and is considered a potential strategy to improve acoustic field uniformity and enhance drying performance [27,28]. MFUS generates a composite acoustic field within the material by simultaneously or alternately emitting two or more ultrasonic frequencies, inducing more intense, sustained, and diverse cavitation effects [29]. Compared with the localized effects of SFUS, the multi-frequency field offers broader frequency coverage and induces multi-scale tissue disturbances across different spatial domains, promoting deep moisture migration while avoiding surface overheating or structural damage [30,31]. Studies have shown that MFUS can achieve synergistic effects of low-frequency penetration and high-frequency agitation through interlaced resonance and frequency superposition, thereby improving energy distribution and utilization in structurally complex samples and more effectively accelerating internal moisture release and diffusion [32]. Existing studies have shown that MFUS can effectively promote cell wall disruption, enhance moisture migration, and help retain bioactive compounds during the drying of fruits, vegetables, and medicinal materials. The dynamically unstable cavitation environment generated by MFUS more readily induces transient high-energy microjets and microscale disturbances, enhancing local mass transfer at the liquid–solid interface and facilitating the elimination of internal moisture migration bottlenecks, thereby improving drying efficiency while reducing energy consumption [33,34]. In summary, the application of MFUS as an enhancement technique in the dehydration of *Cornus officinalis* not only compensates for the limitations of single heat sources or single-frequency acoustic fields, but also enables synergistic optimization of drying efficiency and quality control through multi-scale mass transfer enhancement mechanisms.

At present, research on the mechanism of multi-frequency ultrasound-assisted vacuum far-infrared (MFUS-VFIR) drying remains in its early stages. Existing studies mainly focus on the effects of SFUS on drying rate or quality parameters, while the drying behavior and quality evolution under multi-frequency coupling conditions have not been thoroughly investigated. This study aims to examine the effects of edible coating combined with MFUS-VFIR drying on the drying characteristics and physicochemical properties of *Cornus officinalis*, with emphasis on the variation patterns of drying kinetics, physicochemical attributes, microstructure, and sensory properties under different frequency combinations. The objective is to elucidate the coupling mechanism between heat and mass transfer and quality responses under MFUS. The findings are expected to provide theoretical support and technical guidance for high-quality *Cornus officinalis* processing, as well as new insights for the application of MFUS combined with non-thermal drying technologies in agricultural product processing.

## 2 Materials and methods

### 2.1 Experimental materials

**Fig. 1:**
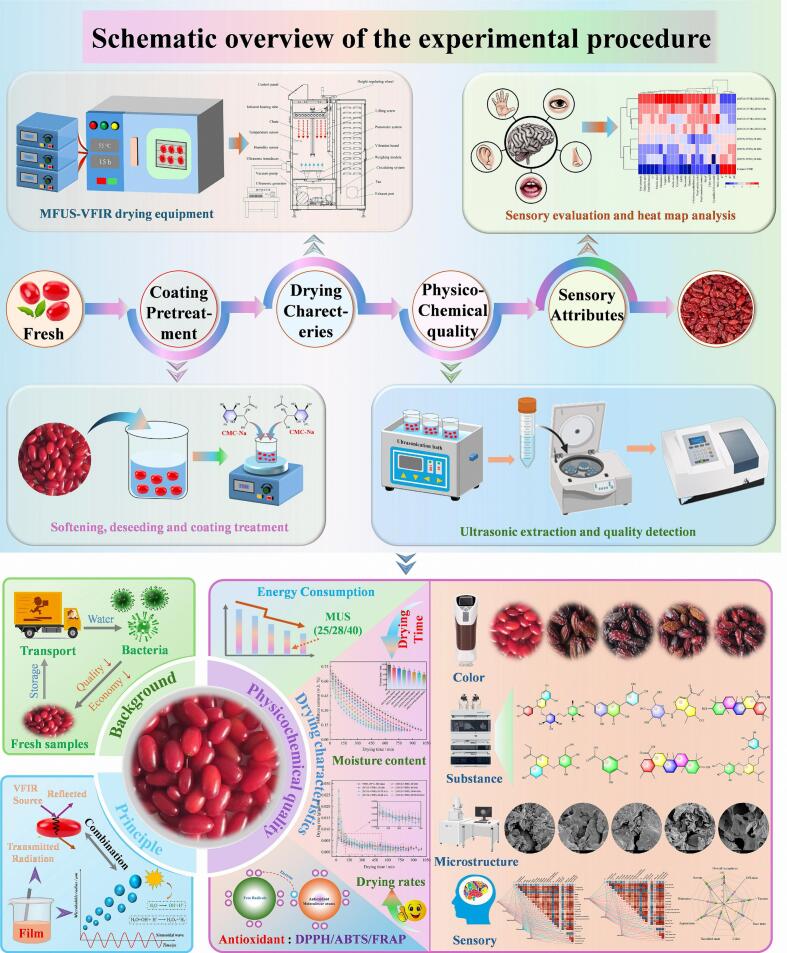
The schematic diagram of multi-frequency ultrasound combined with vacuum far-infrared drying test of *Cornus officinalis*.

The *Cornus officinalis* used in this experiment were harvested from the Foping County, Hanzhong City, Shaanxi Province, China, an area characterized by a superior ecological environment. To ensure sample uniformity and representativeness, naturally ripened, disease-free fresh fruits were selected as raw materials. After harvest, the fruits were manually pre-sorted, and only those with intact appearance, bright red color, smooth skin, uniform size (diameter 15–18 mm), and plump flesh were retained for subsequent experiments. All samples were stored at 4 °C to prevent moisture loss and quality deterioration that could affect experimental outcomes. The flow of the experiment is shown in Fig. 1.

### 2.2 Coating pretreatment

After washing, fresh *Cornus officinalis* fruits were blanched in distilled water at 80 °C for 2  min to soften the flesh, followed by rapid cooling in cold water to room temperature to avoid thermal damage. The cooled fruits were manually halved to remove the seeds, and the deseeded flesh was immediately immersed in a color-preserving solution containing 0.5 % citric acid and 0.2 % ascorbic acid to inhibit enzymatic browning and stabilize color. After color preservation, the samples underwent coating treatment: a 0.5 % (w/v) sodium carboxymethyl cellulose (CMC-Na) solution (A 0.5 % (w/v) CMC-Na solution was prepared by gradually dissolving 0.5 g of CMC-Na in 100 mL of deionized water under continuous magnetic stirring until complete dissolution, followed by degassing to remove air bubbles) was applied uniformly to the fruit surface by spraying. The coated samples were then left to stand in a ventilated, shaded area for 10  min to form a uniform and continuous protective film, providing structural stability and component retention during subsequent drying.

### 2.3 Drying pretreatment

#### 2.3.1 Control experiments

Color-protected fresh *Cornus officinalis* (250.0 ± 1.0 g) were evenly spread in a single layer on a stainless steel tray and subjected to dehydration using VFIR drying equipment (WSH-60A, Shenghua Microwave Technology Co., Ltd, Tianshui, China). Key parameters for VFIR drying were set as follows: drying temperature at 55 °C, distance between the far-infrared radiation source and the sample at 300  mm, and system vacuum pressure maintained at –0.065  MPa. Every 30 min, the sample tray was removed, and the *Cornus officinalis* were weighed using an electronic balance with an accuracy of ± 0.01 g. Each weighing process was completed within 15 s to minimize interruption, after which the tray was promptly returned to the chamber. Drying was finished when the *MC* reached 10 % (w. b).

#### 2.3.2 Single-frequency ultrasound-assisted vacuum far-infrared drying experiments (SFUS-VFIR)

Fresh *Cornus officinalis* samples (250.0 ± 1.0 g) were evenly distributed in a single layer on a stainless steel tray and dehydrated using ultrasound (KM-615B, Kemeng Ultrasonic Instruments Co., Ltd. Zhejiang, China) assisted VFIR drying equipment. Three SFUS modes (20  kHz, 28  kHz, and 40  kHz) were applied separately at a power of 120  W, operated simultaneously with the VFIR process until the moisture content reached 10 % (w. b). All VFIR parameters were identical to those used in the control experiment.

#### 2.3.3 Multi-frequency ultrasound-assisted vacuum far-infrared drying experiments (MFUS-VFIR)

Drying was performed using dual-frequency modes (28/40  kHz, 20/40  kHz, and 20/28  kHz) and triple-frequency mode (20/28/40  kHz). Frequency combinations were realized by synchronous excitation through multiple transducers to achieve resonant superposition, thereby enhancing spatial uniformity and energy transfer efficiency of the ultrasonic field. All other procedures were consistent with those used in the SFUS-VFIR process.

### 2.4 Moisture content (*MC*)and drying rate (*DR*)

The *MC* and *DR* were calculated according to the Eqs. (1), (2) [35]:

(1) $$\mathrm{MC}=\left(M-M_{d}\right)/M$$

(2) $$\mathrm{DR}=\left(M_{t}-M_{t-\Delta t}\right)/\Delta t$$

where *M*_t_ is the dry base moisture content at a specific time, g/g; *M*_d_ is the absolutely dried sample, g/g; *M*_t_ and *M_t-_*_Δt_ are the moisture contents on dry basis at time t and t-Δt, g/g.

### 2.5 Energy consumption

The dehydration of *Cornus officinalis* was conducted using an ultrasound-assisted vacuum far-infrared drying system. Total energy consumption comprised two components: energy used for ultrasound generation and that consumed by the vacuum far-infrared dryer [36]. Unit energy consumption during dehydration was calculated according to Equation (3).

(3) $$\mathrm{TEC}=EC_{\left(US\right)}+EC_{\left(VFIR\right)}$$

where *TEC* denotes the total energy consumption, kW·h·kg^−1^; *EC*_(US)_ represents to the energy consumption generated during ultrasound operation, kW·h·kg^−1^; *EC*_(VFIR)_ represents the energy consumption arising from VFIR operation, kW·h·kg^−1^.

### 2.6 Color measurement

To evaluate the effects of different drying methods on the color characteristics of *Cornus officinalis*, a portable colorimeter (CR-10, Konica Minolta Co., Ltd., Tokyo, Japan) was used to measure the surface color of dried samples [37]. Measurements were performed in the CIELab color space, where L* indicates lightness, a* the red–green, and b* the yellow–blue. For each group, ten uniformly dried and undamaged fruits were randomly selected. Color was measured three times on the same side of each fruit, and the average was calculated to obtain L*, a*, and b* values. Using fresh *Cornus officinalis* as reference, the total color difference (ΔE) and chroma (C) were calculated as follows:

(4) $$\Delta E=\sqrt{{\left(L*-L_{0}\right)}^{2}+{\left(a*-a_{0}\right)}^{2}+{\left(b*-b_{0}\right)}^{2}}$$

(5) $$C=\sqrt{{\left(a*\right)}^{2}+{\left(b*\right)}^{2}}$$

where *L*_0_, *a*_0_, *b*_0_ were the same for fresh *Cornus officinalis*.

### 2.7 Determination of total carotenoids (TCC) and ascorbic acid (AAC)

TCC was measured following the hexane/acetone/methanol extraction method described by Zhang et al. [5] and Santos et al. [38]. Specifically, 0.5  g of dried sample powder was extracted with a mixture of hexane, acetone, and methanol (2:1:1, v/v/v, containing 0.1 % butylated hydroxytoluene). The mixture was vortexed in the dark for 30  min, followed by centrifugation to collect the supernatant. The organic phase was washed with distilled water to remove polar impurities, dehydrated with anhydrous sodium sulfate, and concentrated by rotary evaporation. Absorbance was measured at 450  nm using a spectrophotometer (Shanghai Mapada Instrument Co., Ltd. Shanghai, China).

ACC was determined with reference to the method of Zhang et al. [5]. A commercial ACC assay kit was used, following the manufacturer’s instructions.

### 2.8 Soluble solids content

Soluble solids content was measured by refractometry (PAL-102S, ATAGO Scientific Instrument Co., Ltd, Tokyo, Japan) to assess the retention of soluble substances after drying [39]. A 1.0  g portion of dried sample from each group was mixed with 10  mL of distilled water and extracted in a 37 °C water bath with shaking for 30  min. The extract was filtered through a 0.45  μm membrane, and the filtrate was analyzed. A digital refractometer was used to record the refractive index at 20 ± 0.5 °C. Results were expressed as mass percentage (%). Each sample was measured in triplicate, and the average value was reported.

### 2.9 Determination of natural bioactive compounds, TPC, TFC, and antioxidant activity

#### 2.9.1 Preparation of extract

To assess the effects of different drying treatments on the natural bioactive components and organic acids in *Cornus officinalis*, representative functional compounds were quantified using HPLC (Agilent 1100, Agilent Technology Co., Ltd, USA) [40]. Dried *Cornus officinalis* were ground and passed through an 80-mesh sieve. Precisely 0.5  g of powder was weighed and immersed in 20  mL of 75 % ethanol in a stoppered conical flask. The mixture was shaken at 180  rpm for 48  h in the dark, followed by ultrasonic extraction (240  W, 40  kHz, 30 °C) in a water bath for 30  min. After centrifugation (10  min, 8000  rpm, 4 °C), the supernatant was collected and brought to a final volume of 25  mL with 75 % ethanol. Extracts were stored at 4 °C for determination of total phenolics (TPC), total flavonoids (TFC), antioxidant activity, organic acids, and bioactive compounds.

#### 2.9.2 Determination of natural bioactive compounds

An Agilent Eclipse XDB-C_18_ column (250  mm × 4.6  mm, 5  μm) was used. The mobile phase consisted of 0.3 % phosphoric acid in water (A) and acetonitrile (B), with the following gradient program: 0–10  min, 7 % B; 10–35  min, 7–15 % B; 35–50  min, 15–30 % B; 50–55  min, 30–7 % B; 55–60  min, 7 % B. Column temperature was maintained at 30 °C; flow rate was 1.0  mL/min; injection volume was 10  μL; detection wavelength was set at 240  nm.

#### 2.9.3 Determination of organic acids

An XSelect HSS T3 column was employed. The mobile phase was composed of 3 % methanol and 0.01  mol/L KH_2_PO_4_ (pH 3.0), using isocratic elution at a flow rate of 0.5 mL/min. Column temperature was maintained at 30 °C. Detection was performed at 210  nm with an injection volume of 10  μL.

#### 2.9.4 Determination of TPC

TPC was measured using the Folin–Ciocalteu method, as described by Salehi et al. [41,42] with minor modifications to suit *Cornus officinalis*. Briefly, 0.45  mL of extract was mixed with 2.0  mL of 10 % Folin–Ciocalteu reagent and 1.0  mL of 7.5 % Na_2_CO_3_ solution. After shaking for 5  min, the mixture was incubated in the dark at 37 °C for 1  h. Absorbance was recorded at 765  nm.

#### 2.9.5 Determination of TFC

For TFC analysis, 1.0  mL of extract was sequentially mixed with 2.0  mL of deionized water and 0.3  mL of 5 % NaNO_2_. After 10  min, 0.3  mL of 10 % AlCl_3_·6H_2_O solution was added. The mixture was left to stand for 5  min, followed by addition of 2.0  mL of 1  mol/L NaOH. After thorough mixing, absorbance was measured at 510  nm.

#### 2.9.6 Analysis of DPPH radical scavenging activity

A 0.2  mL aliquot of extract was added to 3.0  mL of 0.1  mmol/L DPPH methanol solution. The mixture was incubated in the dark for 30  min, and absorbance was measured at 515  nm. A 500  μmol/L methanolic solution of ascorbic acid (90 %) was used as a positive control [42].

#### 2.9.7 Analysis of ABTS radical scavenging activity and ferric reducing antioxidant power (FRAP)

ABTS and FRAP were evaluated according to the method of Zang et al [36,43].

### 2.10 Microstructural observation

The surface microstructure of *Cornus officinalis* subjected to different drying treatments was characterized using scanning electron microscopy (S-4800 N, Hitachi Corporation, Tokyo, Japan), following a modified method based on Llavata et al. [44]. Dried samples were fixed in 2.5 % glutaraldehyde solution for 24 h, rinsed three times with phosphate buffer (0.1 mol/L, pH 6.8), and dehydrated with ethanol. After dehydration, samples were freeze-dried for 15 h, gold-coated using a vacuum ion sputter coater. And the surface and internal structure of the samples were observed at × 500 and × 400 times, respectively.

### 2.11 Sensory evaluation

A semi-trained panel sensory evaluation was conducted to assess the effects of different drying methods on the sensory attributes of *Cornus officinalis* [45]. The panel consisted of 25 postgraduate students (13 males and 12 females, aged 20–30) majoring in food science, all with basic sensory evaluation experience and having completed at least 10 h of sensory training to ensure consistency and sensitivity. Evaluations were performed in a standard sensory evaluation room maintained at 25 ± 1 °C, under neutral lighting and odor-free conditions. A 10-point hedonic scale (1 = extremely poor, 10 = excellent) was used to evaluate nine key attributes. Samples were randomly coded and presented in random order, with approximately 5  g of dried fruit provided per evaluation and a 30  s interval between samples. Three independent batches were assessed for each drying method, and average scores were used as final results.

### 2.12 Statistical analysis

All data were expressed as mean ± standard deviation (SD) based on triplicate measurements. Statistical significance was determined using one-way ANOVA followed by Duncan’s multiple range test (*P* < 0.05), performed in SPSS 26.0 (IBM, USA). Additionally, all graphs and data visualizations were generated using OriginPro 2022 to facilitate interpretation and presentation of results.

## 3 Results and discussion

### 3.1 Drying characteristics

**Fig. 2:**
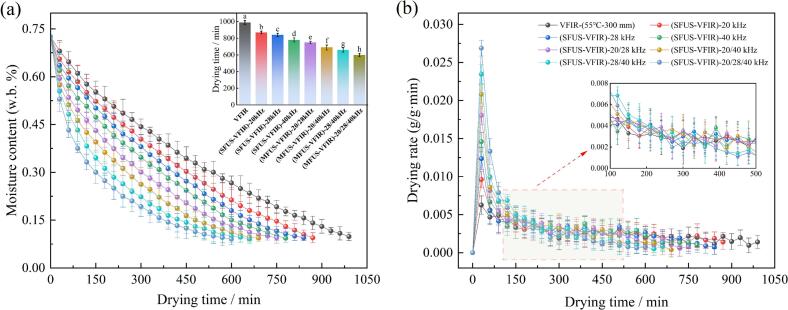
Changes of MC (a) and DR (b) of *Cornus officinalis* under VFIR, SFUS-VFIR, and MFUS-VFIR treatments.

Drying time and drying rate are key indicators of drying efficiency, directly reflecting the kinetics of heat and mass transfer. The drying characteristics of *Cornus officinalis* under single-frequency ultrasound (SFUS) and multi-frequency ultrasound (MFUS) are shown in Fig. 2. After SFUS-VFIR treatments at 20 kHz, 28 kHz, and 40 kHz, the drying times were 870 min, 840 min, and 780 min, respectively, representing reductions of 120 min, 150 min, and 210 min compared to VFIR alone (990 min). Correspondingly, the drying rates increased to 0.30 g/g·min, 0.31 g/g·min, and 0.33 g/g·min, showing enhancements of 15.38 %, 19.23 %, and 26.92 % over VFIR, respectively. These results indicate that ultrasound effectively accelerates moisture migration, thereby improving drying rate and shortening total drying time. This enhancement is primarily attributed to acoustic cavitation, microstreaming, and microscale perturbations generated during ultrasound propagation. The collapse of cavitation bubbles formed in the liquid phase releases localized high energy, inducing instantaneous shear forces and pressure fluctuations that loosen cell walls and enhance intercellular connectivity. This promotes the formation of more continuous internal moisture diffusion channels and reduces resistance to water migration [46].

Further analysis revealed that dual-frequency ultrasound significantly outperformed SFUS treatments in drying efficiency. Drying times under the 20/28 kHz, 20/40 kHz, and 28/40 kHz combinations decreased to 750 min, 690 min, and 660 min, respectively, representing reductions of 3.85 % to 24.13 % compared to those under SFUS-VFIR. The triple-frequency mode (20/28/40 kHz) exhibited the highest drying efficiency, with a total drying time of 600 min. Compared to VFIR and SFUS-VFIR, its drying rate increased by 69.23 % and 33.33–46.67 %, respectively. Studies indicate that SFUS yields a relatively focused acoustic field distribution, often resulting in shadow and standing wave zones. This limits enhancement to localized regions. In dense or structurally heterogeneous *Cornus officinalis* fruit, limited acoustic penetration depth further restricts continuous moisture channel formation. In contrast, MFUS enhances cavitation bubble generation frequency and distribution through composite frequency resonance, forming wider and intersecting microflow pathways between cells. This increases the probability of cell wall rupture and intercellular moisture permeability, facilitating rapid internal moisture migration to the surface [47]. Moreover, different frequencies have distinct cavitation thresholds and intensities; their coupling increases bubble dynamic complexity, sustaining high-intensity cavitation and reducing rapid acoustic energy attenuation [48]. Additionally, synergistic excitation among multiple frequency bands improves acoustic field uniformity. Notably, while MFUS enhances cavitation, inappropriate frequency selection may cause destructive interference within the acoustic field, thereby weakening cavitation intensity. For instance, cavitation under 28/40 kHz was stronger than under 20/28 kHz and 20/40 kHz. In the 20/40 kHz mode, interference from high-frequency components attenuated low-frequency oscillations, reducing cavitation strength in some regions.

### 3.2 Energy consumption

**Fig. 3:**
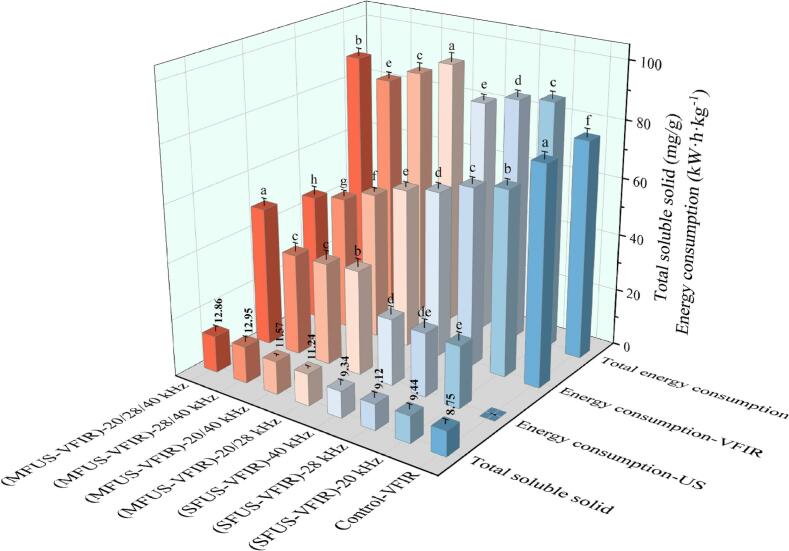
Results of energy consumption and soluble solids content of *Cornus officinalis* under different drying processes.

Energy consumption during drying is a key metric for evaluating process efficiency and industrial applicability. As shown in Fig. 3, the energy consumption of VFIR drying was 76.5 kW·h·kg^−1^. In comparison, SFUS-VFIR treatments at 20, 28, and 40 kHz resulted in slightly higher values, ranging from 81.90 to 87.00 kW·h·kg^−1^. Dual-frequency combinations (20/28 kHz, 20/40 kHz, 28/40 kHz) exhibited energy consumption between 82.95 and 93.00 kW·h·kg^−1^, with the 20/28 kHz mode reaching the highest at 93.00 kW·h·kg^−1^. Although coupling VFIR with ultrasound increased overall energy input, significant differences were observed among frequency combinations, mainly due to variations in acoustic energy distribution, transmission efficiency, and the interplay between acoustic effects and heat–mass transfer. Energy consumption under the integrated (MFUS-VFIR)-20/28/40 kHz treatment decreased to 88.68 kW·h·kg^−1^, which was lower than that of certain dual-frequency modes and comparable to mid-to-high intensity single-frequency treatments. This was attributed to more uniform cavitation and micro-disturbance effects across the *Cornus officinalis*, avoiding energy redundancy and thermal losses caused by single or closely spaced frequencies. Consequently, heat and mass transfer were more efficient, leading to a moderate reduction in specific energy consumption. Notably, although MFUS enhances drying rate and quality retention, its energy-saving performance remains suboptimal. Future research should focus on the role of acoustic field regulation, energy transfer pathways, and the spatiotemporal dynamics of heat–mass exchange to achieve simultaneous optimization of efficiency, quality, and energy economy.

### 3.3 Color

Color is a critical indicator of both the drying quality and sensory acceptance of *Cornus officinalis*, reflecting structural integrity and the retention of pigment-related bioactives (e.g., polyphenols, carotenoids, and anthocyanins) [49]. Color changes during drying are primarily influenced by non-enzymatic browning, polyphenol oxidation, and thermal degradation of heat-sensitive pigments. Table 1 presents the color variations of *Cornus officinalis* under different drying conditions. The control group exhibited the lowest lightness (L*) value (25.38 ± 2.89), indicating pronounced darkening in the absence of ultrasound. This may be attributed to prolonged exposure to high temperature and humidity, leading to heat accumulation and surface oxidation [50]. In the dual-frequency groups, L* increased to 31.44 ± 2.97 under 28/40  kHz, while the 20/28/40  kHz group reached the highest L* value (32.45 ± 3.04), significantly higher than other treatments (*P* < 0.05). This suggests that MFUS-VFIR enhanced internal moisture migration and thermal uniformity, thereby mitigating pigment degradation and reducing brightness loss caused by optical changes during drying. The a* value was highest in the control group (33.84 ± 2.51), indicating increased red hue likely due to accumulation of non-enzymatic browning products. In contrast, dual-frequency treatments showed a* values of 23.87 ± 1.59 to 26.42 ± 2.01, and the triple-frequency group showed a value of 22.85 ± 2.66. These results indicate that MFUS not only delayed browning but also moderately enhanced red saturation, preserving the intrinsic chromaticity of the fruit. This effect may be related to uniform micro-disturbance induced by multi-frequency acoustic fields, promoting stable anthocyanin release and reducing localized overheating. Differences in b* values among groups were relatively minor, yet multi-frequency ultrasound maintained higher yellow saturation, indicating better retention of flavonoid and carotenoid pigments, which was consistent with subsequent physicochemical measurements. The total color difference (ΔE), reflecting overall chromatic changes during drying, showed trends similar to L*. Similar results were reported during multi-frequency power ultrasound of pineapple slices [47]. This is attributed to the cumulative acoustic energy in multi-frequency modes, which triggered complex bubble dynamics and enhanced intercellular mass transfer, facilitating stable pigment preservation. Moreover, the accelerated drying rate reduced total drying time and high-temperature exposure, limiting oxidation and enzymatic reactions [51]. As a result, samples treated with MFUS-VFIR exhibited superior color quality.

**Table 1:** Color, TCC and ACC of dried *Cornus officinalis* products under different drying conditions.

Drying conditions	L*	a*	b*	*ΔE*	*C*	TCC (μg/g)	ACC (mg/g)
Fresh	40.84 ± 3.77^a^	24.08 ± 1.39^f^	3.25 ± 0.12^h^	—	—	21.43 ± 1.24^a^	9.42 ± 0.67^a^
Control-VFIR	25.38 ± 2.89^f^	33.84 ± 2.51^a^	13.44 ± 0.75^a^	20.93 ± 2.10^a^	36.41 ± 2.42^a^	13.01 ± 0.97^f^	5.07 ± 0.54^g^
(SFUS-VFIR)-20 kHz	28.84 ± 1.97^d^	30.88 ± 2.22^b^	10.27 ± 0.67^c^	15.48 ± 2.07^b^	32.54 ± 2.57^b^	13.88 ± 0.88^e^	6.07 ± 0.34^e^
(SFUS-VFIR)-28 kHz	29.48 ± 2.08^cd^	28.29 ± 1.67^c^	9.57 ± 0.45^d^	13.66 ± 1.97^c^	29.86 ± 1.67^c^	14.57 ± 0.75^d^	5.67 ± 0.61^f^
(SFUS-VFIR)-40 kHz	27.65 ± 3.03^e^	27.55 ± 1.92^d^	11.28 ± 1.01^b^	15.83 ± 2.04^b^	29.77 ± 1.97^c^	13.21 ± 0.59^ef^	5.77 ± 0.41^f^
(MFUS-VFIR)-20/28 kHz	29.89 ± 2.55^c^	25.87 ± 2.37^e^	8.54 ± 0.67^e^	12.29 ± 1.67^cd^	27.24 ± 2.64^d^	15.49 ± 0.86^c^	6.97 ± 0.35^d^
(MFUS-VFIR)-20/40 kHz	30.59 ± 2.64^c^	26.42 ± 2.01^d^	7.08 ± 0.94^f^	11.19 ± 1.55^d^	27.35 ± 2.07^d^	15.77 ± 0.94^c^	7.67 ± 0.24^c^
(MFUS-VFIR)-28/40 kHz	31.44 ± 2.97^bc^	23.87 ± 1.59^f^	6.55 ± 0.33^f^	9.96 ± 2.31^e^	24.75 ± 1.57^e^	16.48 ± 0.62^bc^	8.07 ± 0.37^bc^
(MFUS-VFIR)-20/28/40 kHz	32.45 ± 3.04^b^	22.85 ± 2.66^g^	4.87 ± 0.42^g^	8.63 ± 2.42^f^	23.36 ± 2.53^f^	17.09 ± 1.01^b^	8.54 ± 0.40^b^

### 3.4 Total carotenoids (TCC) and ascorbic acid (AAC)

During the drying of *Cornus officinalis*, TCC and AAC, as heat-sensitive and easily oxidized nutrients, are key indicators of nutritional quality and commercial value. As shown in Table 1, drying conditions significantly affected the retention of both compounds (*P* < 0.05), underscoring the critical role of process parameters in maintaining functional component stability. The highest TCC was observed in *Cornus officinalis* treated with (MFUS-VFIR)-20/28/40 kHz, reaching 17.09 ± 1.01  μg/g, which was 1.32 times and 1.17–1.29 times higher than those of the control and SFUS-VFIR, respectively. Carotenoid degradation during dehydration is primarily driven by thermal oxidation, photo-induced breakdown, and lipid peroxidation. Although VFIR provides effective volumetric heating, prolonged exposure to high temperature and humidity may compromise tissue integrity, accelerating carotenoid loss. In contrast, ultrasound promotes rapid moisture gradient formation between internal and external tissues, facilitating surface crust formation that limits oxygen contact and suppresses reactive oxygen species (ROS) generation [52]. Additionally, microturbulence from cavitation may create physical barriers around ester bonds and ring structures in carotenoids, indirectly enhancing molecular stability. No significant differences were found in TCC among dual-frequency groups (*P* > 0.05), ranging from 15.49 ± 0.86 to 16.48 ± 0.62  μg/g.

AAC in samples treated with VFIR, SFUS-VFIR (20  kHz, 28  kHz, and 40  kHz) was 5.07 ± 0.54  mg/g, 6.07 ± 0.34  mg/g, 5.67 ± 0.61  mg/g, and 5.77 ± 0.41  mg/g, respectively, showing reductions of 46.18 %, 35.56 %, 39.81 %, and 38.75 % compared to fresh samples (9.42 ± 0.67  mg/g). Notably, MFUS-VFIR treatments led to a 14.83–42.33 % improvement in AAC retention over SFUS-VFIR, indicating superior protection of heat-labile nutrients. This may result from the synergistic mass and heat transfer effects under multi-frequency ultrasound, which reduce the rate of temperature rise and duration of high-temperature exposure, mitigating thermal degradation. Furthermore, overlapping acoustic waves generate more uniform pressure fields and induce nonlinear bubble dynamics, enhancing microjets and shear forces that disrupt cell wall structures. This facilitates intracellular moisture diffusion, while simultaneously restricting oxygen infiltration and free radical chain reactions, thereby slowing enzymatic oxidation of AAC. In contrast, SFUS often induces localized acoustic pressure concentration and field non-uniformity, leading to uneven moisture migration, edge overheating, or partial oxidation, causing irreversible degradation of AAC in specific regions. Consistently, the highest AAC retention was also observed in samples treated with (MFUS-VFIR)-20/28/40  kHz.

### 3.5 Soluble solids content (SSC)

As a critical parameter indicative of flavor-related compound enrichment, soluble solids content (SSC) reflects the dynamic interaction between mass transfer and energy flow during drying. SSC increased significantly following SFUS-VFIR and MFUS-VFIR treatments, with notable differences among frequency combinations (*P* < 0.05). Compared to VFIR, SSC in samples treated with SFUS-VFIR at 20, 28, and 40  kHz increased by 7.89 %, 4.23 %, and 6.74 %, respectively, indicating enhanced retention due to ultrasound application (Fig. 3). This enhancement is attributed to the concentration effect induced by continuous water diffusion and evaporation during drying. SFUS improves moisture migration, shortens high-temperature exposure time, and reduces degradation risk of heat-sensitive solubles, especially low-molecular-weight sugars and water-soluble acids [53]. Moreover, cavitation-induced microjets and microturbulence mitigate tissue collapse and cementation, reducing secondary diffusion and adhesion of solutes at the cell surface, thereby minimizing SSC loss. A similar observation was highlighted in cherry [36]. However, SFUS is limited by acoustic attenuation and uneven energy distribution, leading to localized effects and reduced enhancement in deeper tissues. In contrast, MFUS-VFIR exhibited superior performance, with SSC values of 11.24 ± 0.77 % and 11.57 ± 0.67 % under 20/28  kHz and 20/40  kHz, respectively. The 28/40  kHz combination further increased SSC to 12.95 ± 1.01 %, demonstrating a synergistic effect of MFUS in promoting SSC accumulation. Under multi-frequency resonance, the spatial distribution of acoustic energy is more uniform, avoiding rapid surface heating and structural densification caused by local energy overlap, and reducing thermal degradation risk. This facilitates the release of intracellular soluble sugars and flavor precursors, enhancing SSC of *Cornus officinalis*. Notably, SSC in *Cornus officinalis* samples treated with (MFUS-VFIR)-20/28/40  kHz reached 12.86 ± 0.97 %, significantly higher than that of VFIR and SFUS-VFIR (*P* < 0.05), though slightly lower than the 28/40  kHz group. This may be due to more dispersed energy under triple-frequency conditions, resulting in slightly reduced disturbance in intracellular fluid-rich regions and thus marginally lower SSC.

### 3.6 Natural bioactive compounds

**Fig. 4:**
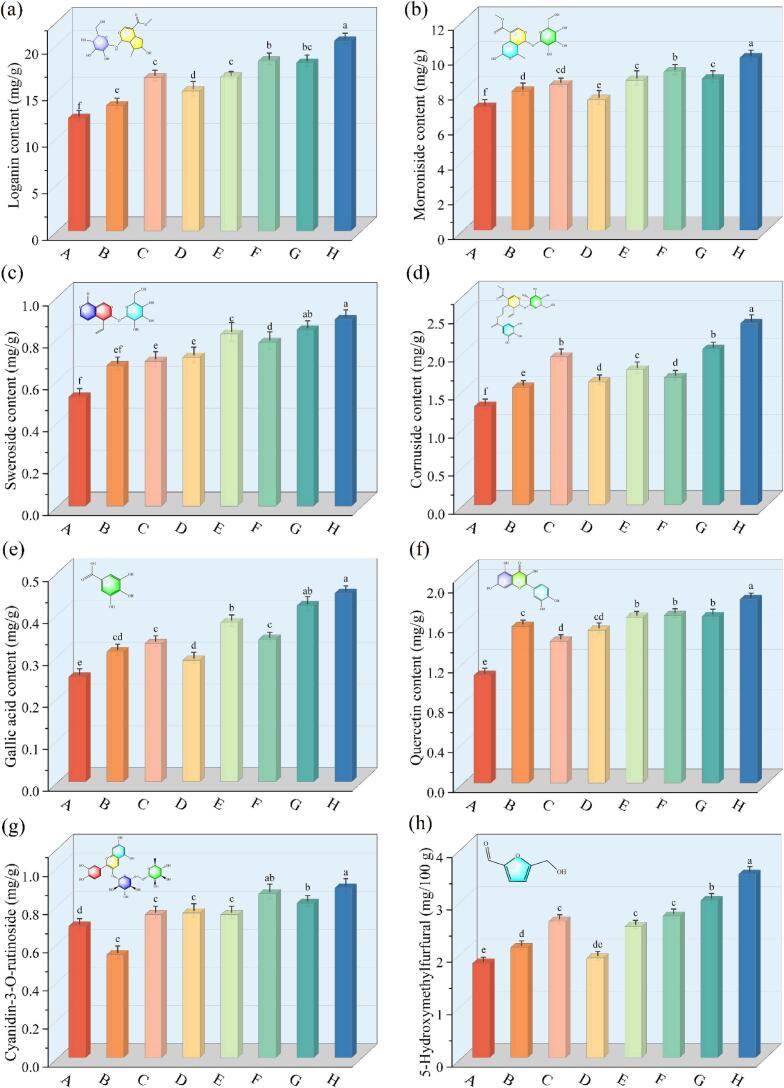
Content of natural active compounds in *Cornus officinalis* after VFIR, SFUS-VFIR and MFUS-VFIR treatments.

During the drying of *Cornus officinalis*, the stability and retention of natural bioactive compounds serve as key indicators of drying quality. Particularly important are iridoids, phenolic acids, flavonoids, and anthocyanins (cyanidin-3-O-rutinoside), which are critical to the dual medicinal and nutritional value of *Cornus officinalis* [54]. Changes in their contents under different drying conditions are shown in Fig. 4. Under SFUS-VFIR, samples treated at 28 kHz exhibited higher retention of active compounds compared to those at 20 kHz and 40 kHz. Specifically, the contents of loganin, morroniside, and cornuside were 16.44 ± 0.57 mg/g, 8.34 ± 0.24 mg/g, and 1.94 ± 0.14 mg/g, respectively; gallic acid and 5-hydroxymethylfurfural reached 0.33 ± 0.02 mg/g and 2.60 ± 0.07 mg/g. Compared with 20 kHz and 40 kHz, these values increased by 22.32 %, 4.77 %, 25.97 %, 6.45 %, 23.81 %, and 9.52 %, 11.50 %, 20.50 %, 13.79 %, 36.84 %, respectively. Dual-frequency treatments further improved retention, with the 28/40 kHz combination showing the highest levels of sweroside and gallic acid at 0.84 ± 0.03 mg/g and 0.42 ± 0.06 mg/g. This effect is attributed to the frequency complementarity of the composite acoustic field: high-frequency waves enhance surface permeability, while mid-frequency waves facilitate disruption of internal cellular structures, together reducing diffusion resistance and degradation risks. Although 20/40 kHz and 20/28 kHz combinations also outperformed single-frequency treatments, their retention effects were suboptimal, suggesting that the efficiency of dual-frequency synergy remains constrained by the matching compatibility of frequency ratio and wavelength. Under (MFUS-VFIR)-20/28/40 kHz, most active compounds reached their highest retention. For instance, loganin, sweroside, and morroniside contents were 20.37 ± 0.59 mg/g, 0.89 ± 0.07 mg/g, and 9.88 ± 0.31 mg/g, respectively, significantly higher than other treatments (*P* < 0.05). This indicates that the multi-frequency composite acoustic field enables multiscale and multidirectional energy transmission pathways, enhancing the acoustic response area within intercellular and intracellular structures. It also induces a dynamic non-steady-state environment that facilitates selective cellular disruption and reconstruction of permeation channels, thereby improving intracellular compound release. Moreover, MFUS-VFIR enhanced the driving force for intracellular component migration, reduced energy resistance at diffusion interfaces, and prevented oxidation and degradation caused by localized energy concentration. The stabilizing effect on thermolabile compounds such as cyanidin-3-O-rutinoside and gallic acid was particularly pronounced due to more uniform energy distribution and shorter exposure cycles. This avoided excessive localized sound pressure or energy accumulation observed under single-frequency conditions, thereby suppressing enzyme-catalyzed and free-radical oxidation reactions initiated by metal ions in the microenvironment, and protecting phenolic hydroxyl groups and unsaturated double bonds within molecular structures.

### 3.7 Organic acids

**Fig. 5:**
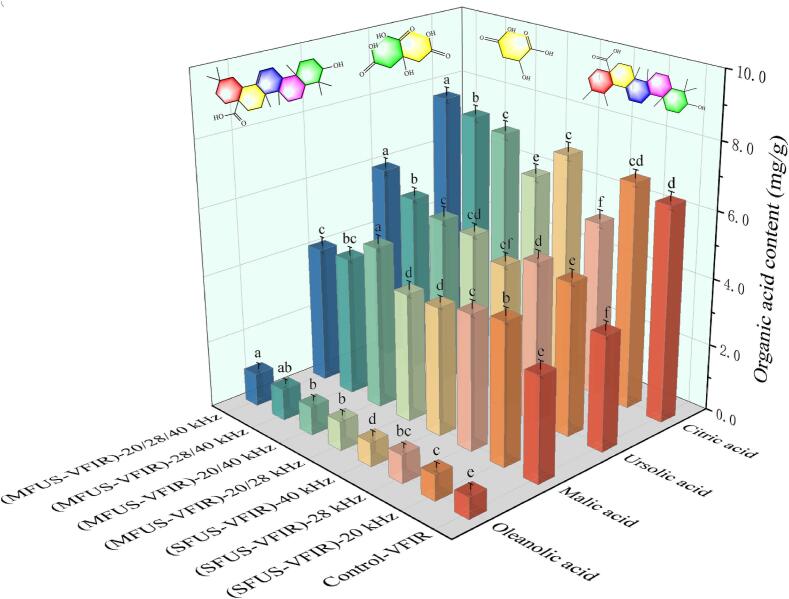
Content of four organic acids in *Cornus officinalis* after VFIR, SFUS-VFIR and MFUS-VFIR treatments.

As key contributors to the flavor and functional activity of *Cornus officinalis*, the content of organic acids is jointly influenced by thermal transfer efficiency, the extent of tissue disruption, and the degradation rate of active compounds. Fig. 5 illustrates the variation in organic acid content under different drying conditions. After VFIR treatment, the retention of malic acid, oleanolic acid, and ursolic acid was generally lower than that observed in SFUS-VFIR and MFUS-VFIR treatments. This reduction is primarily attributed to the prolonged heat exposure during VFIR, which promotes thermal degradation, oxidation, and migration loss of organic acids. In the absence of ultrasound assistance, limited cell wall rupture increases internal diffusion resistance and reduces the drying rate, thereby exacerbating the degradation of thermolabile organic acids.

SFUS-VFIR and MFUS-VFIR treatments increased the contents of oleanolic acid, malic acid, and ursolic acid by 12.50–53.13 %, 21.90–53.97 %, and 25.80–71.30 %, respectively, compared to VFIR. This enhancement is attributed to ultrasonic cavitation and mechanical effects during the early drying stage, which improve the permeability of cell walls and membranes. As a result, both free and bound forms of organic acids are released more gently and can migrate extracellularly at lower temperatures, reducing acid degradation caused by thermal exposure. Additionally, ultrasound may induce the release of endogenous antioxidant enzymes and stress-response compounds, such as CAT and SOD, which can inhibit oxidative degradation of organic acids. Under vacuum, ultrasound further accelerates gas release and tissue collapse, thereby mitigating oxidative stress on thermosensitive components by reducing oxygen availability. After MFUS-VFIR treatment at 20/28/40 kHz, *Cornus officinalis* showed the highest retention of oleanolic acid, ursolic acid, and citric acid, reaching 0.98 ± 0.11 mg/g, 5.91 ± 0.37 mg/g, and 7.59 ± 0.46 mg/g, respectively. However, malic acid was more abundant following (MFUS-VFIR)-20/40 kHz. These results may be explained by the MFUS-induced increase in cavitation bubble generation frequency and spatial distribution, which reduces localized structural damage and enhances cellular integrity, thereby minimizing non-selective exudation of organic acids. Furthermore, energy overlap among frequencies produces more uniform bubble collapse dynamics, preventing localized overheating or violent cavitation, which otherwise accelerates thermal degradation of acidic compounds. This effect contributes to improved stability and retention of organic acids.

### 3.8 Total phenolic (TPC) and total flavonoid contents (TFC)

**Fig. 6:**
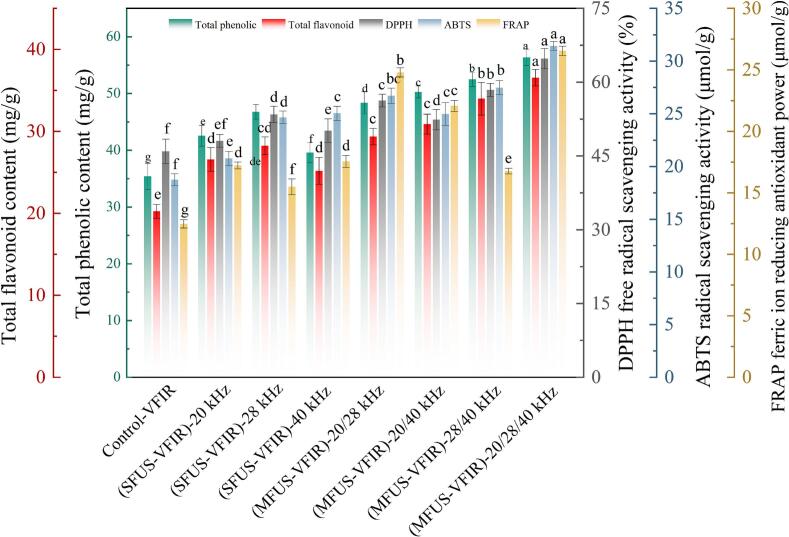
Variation patterns of TPC, TFC and antioxidant capacity (DPPH, ABTS, and FRAP) in dried products of *Cornus officinalis*.

TPC and TFC are principal pharmacologically active constituents of *Cornus officinalis*, extensively involved in its antioxidant, anti-inflammatory, hypoglycemic, and neuroprotective effects [55,56]. Fig. 6 presents the TPC and TFC of dried samples subjected to different dehydration treatments. After VFIR drying, the TPC and TFC were 35.41 ± 1.28 mg/g and 20.25 ± 0.84 mg/g, respectively, representing the lowest values among all treatments. In SFUS-VFIR drying, the 28 kHz condition exhibited superior retention of these compounds, with TPC and TFC reaching 46.77 ± 2.39 mg/g and 28.24 ± 1.07 mg/g, respectively. Compared with the 20 kHz and 40 kHz treatments, these values increased by 9.37 % and 6.25 % for TPC, and by 18.20 % and 12.20 % for TFC, which may be related to differences in acoustic energy transfer efficiency or the depth of sound field penetration. Although VFIR combines the penetration capability of far-infrared radiation with the low-temperature dehydration advantage of a vacuum environment, in dense matrices such as *Cornus officinalis*, it often results in uneven internal heat conduction and hindered moisture migration, leading to localized overheating and accelerated degradation of thermolabile phenolic and flavonoid compounds. The introduction of MFUS improved water diffusion pathways and migration efficiency, shortening heat exposure duration and consequently reducing the probability of oxidative and thermal degradation of TPC and TFC. The highest retentions of TPC (56.35 ± 2.43 mg/g) and TFC (36.54 ± 1.04 mg/g) were observed in (MFUS-VFIR)-20/28/40 kHz drying, with statistically significant differences (*P* < 0.05) compared with VFIR and dual-frequency combinations. This could be attributed to the more complex acoustic field generated by multi-frequency synergy. Triple-frequency ultrasound resonance induced multiscale cavitation within *Cornus officinalis*, enabling cavitation thresholds and bubble dynamics associated with different frequencies to coexist simultaneously, thereby enhancing multipoint rupture of cell walls and middle lamellae, intensifying cellular disintegration, and improving the release efficiency of phenolic and flavonoid compounds. In addition, compared with dual-frequency modes, the triple-frequency mixed ultrasound field exhibited greater uniformity of acoustic energy distribution, effectively avoiding local sound intensity overlap and “dead zones,” and promoting homogeneous structural disruption across the entire sample [57]. This synergistic acoustic effect not only enhanced mass transfer efficiency during drying but also reduced cumulative thermal load and oxidative exposure, thereby mitigating the loss of phenolic and flavonoid compounds due to thermally induced degradation.

### 3.9 Antioxidant capacity

*Cornus officinalis* is rich in natural antioxidant constituents, including polyphenols, flavonoids, and organic acids, whose preservation is closely related to the medicinal and nutritional value of the dried product. Fig. 6 illustrates the variation in antioxidant capacity of dried *Cornus officinalis* under different frequency conditions. Relative to VFIR, ultrasound-assisted VFIR treatments enhanced antioxidant capacity to varying extents, with the most pronounced improvement observed in the multi-frequency group. The conclusion that MFUS treatment can improve the antioxidant capacity of agricultural products was found in the research of Xu et al. [58]. In particular, (MFUS-VFIR)-20/28/40 kHz treatment yielded higher DPPH radical scavenging activity, ABTS radical scavenging activity, and ferric reducing antioxidant power (FRAP), at 64.77 ± 1.79 %, 31.44 ± 0.42 μmol/g, and 26.55 ± 0.38 μmol/g, respectively. Compared with SFUS-VFIR, these values increased by 21.20–53.44 %, 25.56–51.37 %, and 51.28–71.40 %, respectively, indicating that MFUS more effectively preserved or facilitated the release of antioxidants in *Cornus officinalis*. SFUS primarily enhances the formation of cavitation channels within tissues, promoting moisture migration and thereby indirectly reducing thermal exposure during drying, which slows thermal degradation of antioxidant compounds. And due to spatial limitations in acoustic energy distribution and rapid attenuation of cavitation intensity with propagation depth, its enhancement in deeper tissues is constrained. Moreover, cavitation bubble behavior at a fixed frequency is relatively uniform, prone to localized acoustic pressure superposition and temperature rise, which may elevate oxidative stress and compromise the structural stability of antioxidants such as vitamin C and anthocyanins, thereby diminishing antioxidant capacity [59]. In contrast, frequency superposition allows low-frequency ultrasound, with its strong cavitation capacity, to disrupt cellular structures and release intracellular contents, while high-frequency ultrasound induces more intense micro-agitation and permeability changes at tissue surfaces. This synergy enables targeted release and stable diffusion of antioxidant constituents, reducing oxidative loss during migration. Additionally, the non-steady composite acoustic field generated by MFUS facilitates localized energy release in intercellular spaces and near membrane structures, perturbing the dynamic balance of enzymatic systems and metal ions within the cellular microenvironment. This suppresses the propagation rate of free radical chain reactions, helping to maintain the reduced state of hydroxyl groups and unsaturated bonds, thereby enhancing overall antioxidant capacity. In dual-frequency ultrasound treatments, the 20/28 kHz combination produced higher antioxidant capacity (DPPH = 56.24 ± 1.31 %; ABTS = 26.71 ± 0.73 μmol/g; FRAP = 24.79 ± 0.37 μmol/g) than other dual-frequency conditions, while the 28/40 kHz and 20/40 kHz combinations showed comparable but lower values than those achieved by MFUS.

### 3.10 Microstructural characteristics

**Fig. 7:**
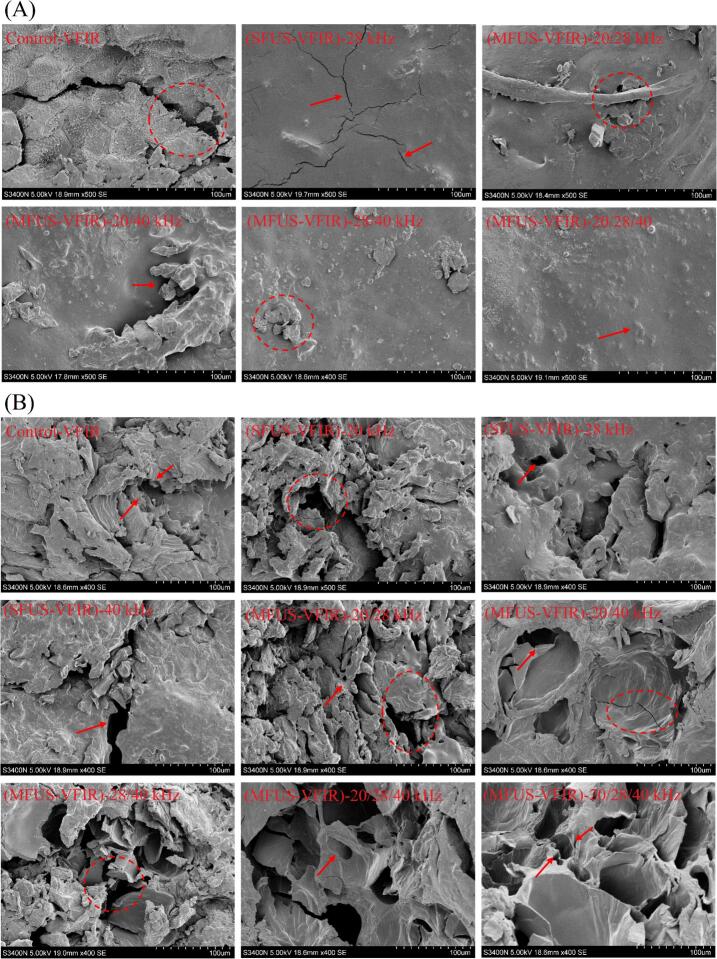
Schematic representation of the surface and internal microstructure of *Cornus officinalis* after different dehydration treatments.

Fig. 7 (A) and (B) present the surface and internal microstructures of *Cornus officinalis* under different drying conditions. In the control group, cells exhibited pronounced collapse, shrinkage, and agglomeration, with indistinct intercellular spaces, rough surfaces, and numerous cracks. These features were likely due to uneven thermal stress during VFIR drying, causing cell wall contraction and rapid surface moisture evaporation, which induced a hardening effect that hindered subsequent internal moisture migration, ultimately forming a dense and sealed internal structure. Samples treated with SFUS-VFIR showed partial loosening of cell walls and localized increases in porosity. Notably, at 28 kHz, surface cracks were reduced, and distinct microchannels and pores appeared within the tissue, indicating that ultrasonic cavitation and microstreaming effectively weakened intercellular adhesion, enhancing tissue dehydratability [60]. However, the fixed frequency led to strong acoustic energy focusing, resulting in localized over-disruption or dead-zone effects, with non-uniform pore distribution and partial collapse remaining. By comparison, dual-frequency groups exhibited a more continuous and well-developed microporous network, with blurred but largely intact cell boundaries, and greater surface uniformity compared to single-frequency treatments. Among these, the 28/40 kHz group displayed the most compact tissue structure with relatively uniform pore size distribution, suggesting that frequency coupling generated multiscale cavitation and shear fields, markedly improving acoustic energy penetration and facilitating balanced heat–mass transfer [61]. Following (MFUS-VFIR)-20/28/40 kHz treatment, *Cornus officinalis* developed a more pronounced porous architecture, with well-organized surface structures, significantly fewer cracks, and cell walls forming an intact honeycomb-like pore pattern. This indicates that in triple-frequency ultrasound, acoustic energy distribution is more uniform and penetration more thorough, enabling synergistic cavitation and vibrational perturbation at multiple acoustic field loci. Such effects promote the formation of optimal multilevel microchannel pathways for moisture migration, facilitating rapid release of bound water from deeper tissues and enhancing heat and mass transfer efficiency, without excessive damage to overall cellular integrity. Furthermore, increasing ultrasound frequency enlarged pore diameter and increased pore number, a phenomenon attributable to intensified mechanical action under multi-frequency operation, which induced repeated expansion and contraction of cellular structures, thereby promoting the formation and enlargement of microchannels.

### 3.11 Sensory properties

**Fig. 8:**
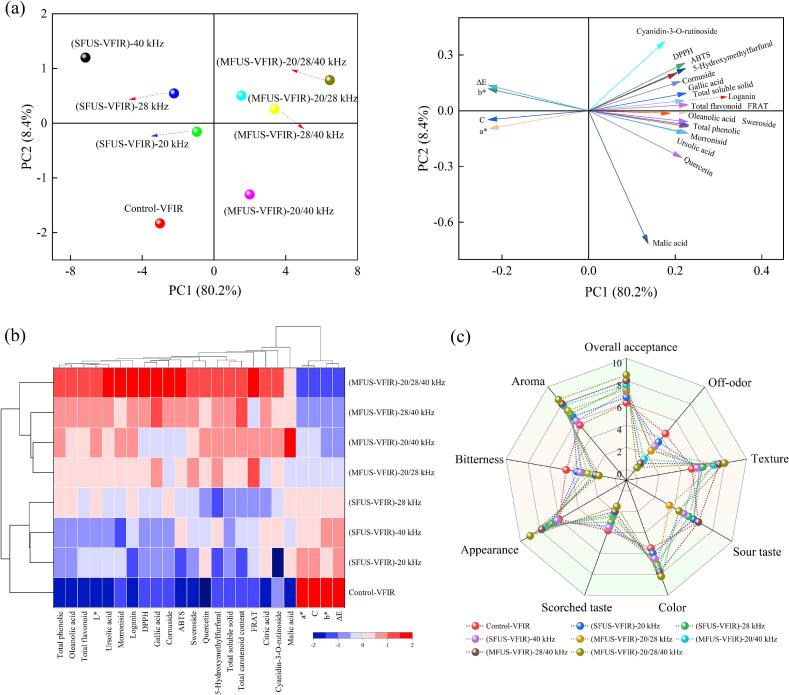
Schematic representation of HCA, PCA and sensory evaluation of Cornus officinalis under different dehydration treatments.

The sensory attributes of *Cornus officinalis* constitute critical indicators for assessing consumer acceptance, encompassing color, appearance, texture, flavor (sourness, bitterness), and overall sensory score. Drying processes exert a pronounced influence on these attributes, particularly given the susceptibility of thermolabile components to degradation and the vulnerability of tissue structures to damage; thus, process selection directly determines the sensory quality of the final product. Sensory evaluation revealed that the control exhibited pronounced textural hardening, darkened color, and intensified bitterness, with an overall acceptance score of only 6.0 (Fig. 8(c)). In contrast, the SFUS-VFIR group improved color uniformity (score increased to 6.0–7.0) and mitigated off-flavor and bitterness. Salehi et al. [62] also reported that the combination of ultrasound with edible coating treatment significantly improved the color quality and sensory attributes of orange slices. However, its texture score remained relatively low (5.5–6.0), with visible surface over-cracking and fibrous flesh, likely associated with uneven local acoustic enhancement and concentrated structural disruption. Dual-frequency treatments markedly enhanced appearance integrity and texture scores (6.2–6.8 and 5.5–6.0, respectively), producing a more natural reddish-brown hue and moderate chewiness, while significantly reducing bitterness and balancing sourness, thereby improving overall flavor harmony. Among them, the 28/40 kHz group (8.0) achieved the highest overall acceptance within the dual-frequency treatments, significantly surpassing SFUS-VFIR and the control (*P* < 0.05). This effect may result from coupled enhancement between the surface and inner tissues under MFUS, accelerating the release of volatile off-flavor compounds while retaining sourness and aromatic flavor constituents [43,63]. Furthermore, the improved drying uniformity achieved by MFUS-VFIR contributed to suppressing epidermal browning and excessive moisture loss in the pulp, thereby maintaining color stability and textural consistency. The (MFUS-VFIR)-20/28/40 kHz samples attained the highest overall acceptance score (8.5 ± 0.15), slightly exceeding the dual-frequency group (7.2–8.0), with the control group scoring lowest. Bitterness and off-flavor scores were also minimal in the MFUS-VFIR treatment. This can be attributed to the non-steady-state perturbation environment induced by triple-frequency ultrasound, which favors the stability of key flavor-active molecules, particularly thermosensitive organic acids and aromatic compounds, thereby preserving the natural flavor profile of *Cornus officinalis* and inhibiting off-flavor formation [57,64].

### 3.12 Principal component analysis (PCA) and hierarchical cluster analysis (HCA)

PCA and HCA were applied to all physicochemical data for dimensionality reduction and similarity grouping, thereby elucidating differences and similarities in sample characteristics under various drying conditions and identifying key influencing factors [65]. PCA results showed that PC1 and PC2 together accounted for 88.6 % of the total variance (Fig. 8(a)). PC1 was primarily driven by total phenolics, total flavonoids, FRAP, soluble solids, morroniside, and organic acids, reflecting antioxidant capacity as well as the enrichment of color and flavor constituents. PC2 was mainly influenced by color difference values, indicating an intrinsic association between color and structural stability. Distribution analysis revealed that samples treated with (MFUS-VFIR)-20/28/40 kHz clustered in the positive quadrant of both PC1 and PC2, showing strong overlap with variables positively associated with quality enhancement. In contrast, single-frequency treatments and the control shifted toward the negative quadrant, indicating certain disadvantages in multidimensional quality performance. Dual-frequency treatments, particularly 28/40 kHz and 20/40 kHz, also clustered in the positive region of PC1 but displayed slight variation along PC2, suggesting differences in structural stability or retention of native bioactive compounds.

HCA results exhibited a clear hierarchical pattern, indicating differences in the response profiles of major quality attributes among drying methods (Fig. 8(b)). Vertically, (MFUS-VFIR)-20/28/40 kHz, (MFUS-VFIR)-20/28 kHz, and (MFUS-VFIR)-28/40 kHz were grouped together, showing comparable and superior performance in retaining carotenoids, native bioactives, antioxidants, and phenolic compounds. This indicates that multi-frequency ultrasound assisted drying produces consistent responses across different quality dimensions, reflecting its composite acoustic field mechanism in protecting thermosensitive components. By contrast, the three SFUS-VFIR treatments and VFIR-dried samples formed another branch, showing marked disadvantages in retaining certain bioactive constituents. This may be attributed to the limitations of SFUS in cavitation intensity, sound pressure distribution, and tissue disruption patterns, resulting in lower mass transfer efficiency and weaker microenvironment regulation compared with multi-frequency systems. Notably, VFIR and (SFUS-VFIR)-20 kHz treatments produced *Cornus officinalis* whose quality indicators deviated substantially from other groups, displaying clustering isolation and further highlighting deficiencies in overall quality maintenance. Horizontal clustering revealed that organic acids, AAC, and native bioactives formed a highly correlated feature set, indicating strong consistency in their responses to acoustic field modulation during drying.

### 3.13 Correlation network heatmap analysis

**Fig. 9:**
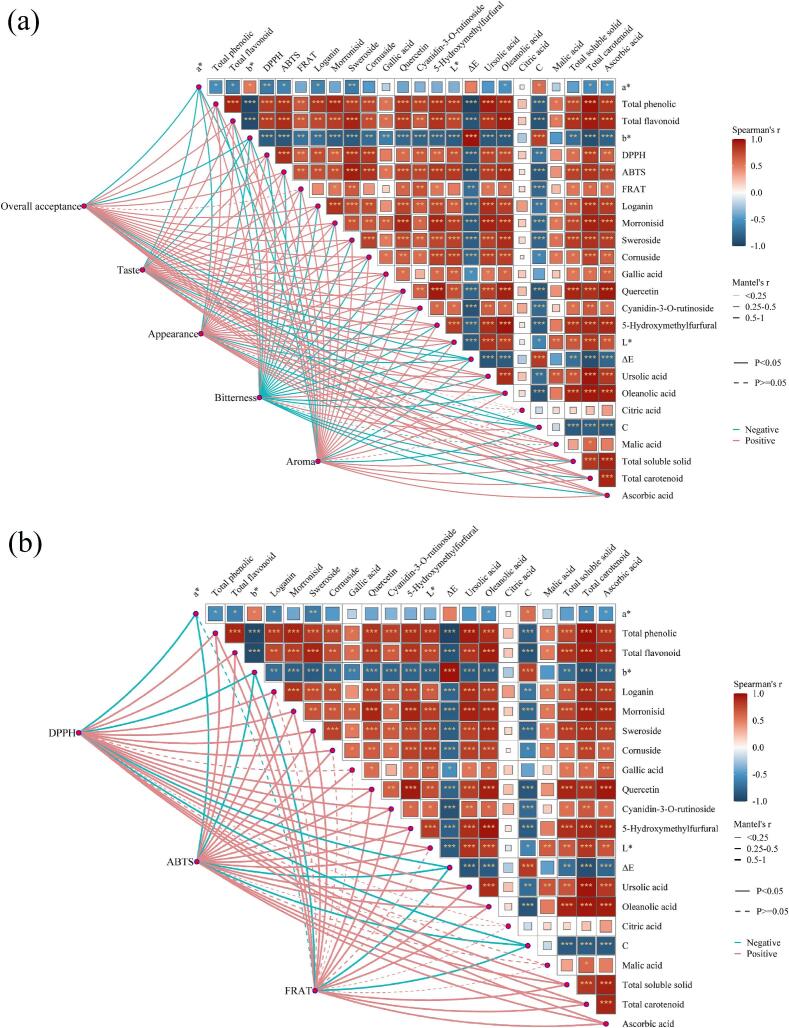
Correlation network heatmap analysis of sensory evaluation and antioxidant capacity.

The correlation network heatmap clearly revealed the intrinsic associations among physicochemical quality parameters of *Cornus officinalis*, providing data support for understanding its quality formation mechanism. Overall, most parameters exhibited significant correlations and strong coupling, indicating that the physicochemical properties of *Cornus officinalis* during drying do not change independently but are mutually synergistic or restrictive. In the diagram, cyan indicates negative correlations and pink indicates positive correlations. The sensory attribute network heatmap showed that overall acceptability of dried *Cornus officinalis* was positively correlated with antioxidant capacity, native bioactive compounds, and organic acids (Fig. 9(a)). Specifically, morroniside, loganin, cornuside, ursolic acid, and oleanolic acid exhibited significant positive correlations with overall acceptability (*P* < 0.05), indicating that iridoids and triterpenoids play a key role in imparting the characteristic flavor and mouthfeel of dried products, possibly by enhancing fruity aroma and balancing acidity–astringency to improve overall sensory perception. Similarly, the influence patterns of physicochemical attributes on aroma and taste were consistent with those on overall acceptability. Notably, among the four organic acids, citric acid had no significant effect on overall acceptability or taste (*P* > 0.05). Color, malic acid, oleanolic acid, and ursolic acid were significantly negatively correlated with bitterness (r > 0.80, *P* < 0.05), likely because organic acids lower the internal pH of *Cornus officinalis*, altering the dissociation state of bitter compounds and thereby reducing their binding affinity to bitterness receptors, which attenuates bitterness intensity. Furthermore, organic acids often impart fruity or refreshing notes, creating a flavor contrast with bitterness and enhancing the perception of other pleasant flavor notes, thus exerting an antagonistic effect on bitterness. This differs from the findings of Zang et al. [57] for cherries, which may be related to the optimization of energy distribution by MFUS during dehydration. The antioxidant property network heatmap revealed positive correlations (r > 0.85, *P* < 0.05) between active components such as TPC, TFC, and anthocyanins with antioxidant capacity indices including DPPH, ABTS, and FRAP, suggesting that the antioxidant activity of *Cornus officinalis* is mainly derived from the synergistic effects of phenolics and flavonoids (Fig. 9(b)). Malic acid and citric acid showed no significant influence on FRAP, ABTS, and DPPH (*P* > 0.05).

## 4 Conclusion

To address the prolonged drying cycle and quality deterioration of *Cornus officinalis* under conventional drying, this study conducted a systematic analysis of drying kinetics, bioactive compound retention, color characteristics, microstructure, sensory attributes, and multidimensional quality evaluation. The results demonstrated that CMC-Na coating combined with MFUS-VFIR drying markedly enhanced heat and mass transfer efficiency and quality stability. MFUS, particularly the (MFUS-VFIR)-20/28/40 kHz treatment, facilitated uniform expansion of internal micropore networks during dehydration, reduced the occurrence of surface cracking, effectively preserved key natural bioactive constituents and intrinsic color integrity, and achieved superior sensory acceptability and flavor balance. Compared with SFUS-VFIR, (MFUS-VFIR)-20/28/40 kHz increased TPC, TFC, soluble solids, TCC, AAC, DPPH, ABTS, and FRAP by 20.48–42.41 %, 29.39–45.17 %, 36.23–41.01 %, 17.30–29.37 %, 40.69–50.62 %, 21.20–34.74 %, 25.56–51.37 %, and 51.28–71.40 %, respectively. Furthermore, dual-frequency ultrasound treatment exerted a positive effect on the retention of bioactive compounds in *Cornus officinalis*, particularly under 28/40 kHz conditions, where superior stability in color retention and overall quality was observed. PCA, HCA, and correlation network heatmap analysis indicated that MFUS-treated samples were highly clustered in the multidimensional quality space and exhibited significant positive correlations with antioxidant capacity and physicochemical parameters. Notably, the energy consumption of this technique was slightly higher than that of VFIR and SFUS-VFIR. Therefore, future work should focus on elucidating the critical role of MFUS in acoustic field parameter modulation, energy utilization pathways, and its effects on the spatiotemporal distribution of heat and mass transfer, to achieve synergistic optimization of drying efficiency, quality preservation, and energy economy. This study provides a theoretical basis for the optimization of energy-efficient drying processes, precise quality control, and industrial application of *Cornus officinalis*.

## CRediT authorship contribution statement

**Zepeng Zang:** Writing – review & editing, Writing – original draft, Validation, Supervision, Methodology, Investigation, Formal analysis, Data curation, Conceptualization. **Xiaopeng Huang:** Writing – review & editing, Visualization, Supervision, Resources, Project administration, Methodology, Funding acquisition, Conceptualization. **Guojun Ma:** Resources, Project administration, Investigation, Conceptualization. **Fangxin Wan:** Visualization, Validation, Methodology, Conceptualization. **Xiaoping Yang:** Methodology, Investigation, Formal analysis. **Qiaozhu Zhao:** Validation, Methodology, Data curation, Conceptualization. **Yanrui Xu:** Visualization, Methodology, Investigation. **Fei Dai:** Writing – review & editing, Resources, Project administration, Formal analysis.

## Declaration of competing interest

The authors declare that they have no known competing financial interests or personal relationships that could have appeared to influence the work reported in this paper.
